# Advances in low-protein diets for swine

**DOI:** 10.1186/s40104-018-0276-7

**Published:** 2018-07-19

**Authors:** Yuming Wang, Junyan Zhou, Gang Wang, Shuang Cai, Xiangfang Zeng, Shiyan Qiao

**Affiliations:** 1 State Key Laboratory of Animal Nutrition, College of Animal Science and Technology, China Agricultural University, Beijing, 100193 China; 2 Beijing Key Laboratory of Biological Feed Additive, China Agricultural University, Beijing, 100193 China

**Keywords:** Amino acid, Crude protein, Growth performance, Gut health, Net energy, Nutrient balance, Pig

## Abstract

Recent years have witnessed the great advantages of reducing dietary crude protein (CP) with free amino acids (AA) supplementation for sustainable swine industry, including saving protein ingredients, reducing nitrogen excretion, feed costs and the risk of gut disorders without impairing growth performance compared to traditional diets. However, a tendency toward increased fatness is a matter of concern when pigs are fed low-protein (LP) diets. In response, the use of the net energy system and balanced AA for formulation of LP diets has been proposed as a solution. Moreover, the extent to which dietary CP can be reduced is complicated. Meanwhile, the requirements for the first five limiting AA (lysine, threonine, sulfur-containing AA, tryptophan, and valine) that growing-finishing pigs fed LP diets were higher than pigs fed traditional diets, because the need for nitrogen for endogenous synthesis of non-essential AA to support protein synthesis may be increased when dietary CP is lowered. Overall, to address these concerns and give a better understanding of this nutritional strategy, this paper reviews recent advances in the study of LP diets for swine and provides some insights into future research directions.

## Background

In pig production, the dietary crude protein (CP) content can be reduced when the requirements for essential amino acids (EAA) and total nitrogen are met, because for pigs the need for dietary protein is essentially a need for amino acids (AA) [1]. Because of limited lysine content in corn, higher amount of soybean meal (SBM) was included in traditional corn-soybean meal (CSBM) diets to meet lysine requirement of pigs, which resulted in high CP levels [2]. High-protein (HP) diets led to excesses of other EAA and excretion of excess nitrogen in feces and urine, resulting in lowering the efficiency of nitrogen utilization. Additionally, protein fermentation in hindgut can damage the gut health. Reducing the dietary CP by 2% to 4% from the NRC (1998) [3] recommendations and supplementing with crystalline amino acids (CAA) has been demonstrated to increase nitrogen utilization, reduce feed costs and nitrogen excretion, and promote gut health without impairing the growth performance of pigs [4–7]. Low-protein (LP) diets in these studies supplemented with four crystalline amino acids (FCAA) (*L*-lysine, *DL*-methionine, *L*-threonine and *L*-tryptophan) because they are the first four limited AA needed to be added to balance for an ideal protein ratio.

The latest NRC (2012) [8] for standard nutrient requirements of swine eliminated the recommendations for CP requirement and replaced it with a total nitrogen requirement. If the total nitrogen requirement of NRC (2012) [8] is multiplied by the common CP coefficient of 6.25, the CP requirement is 2% to 4% lower than the recommended value of the NRC (1998) [3]. This indicated that the study of LP diets had advanced and the advancement could be applied to pig production. With the development of industrial synthetic AA technology, supplementary feed-grade AA, such as *L*-valine and *L*-isoleucine have become available for use in livestock diets, resulting in the potential for further reduction in dietary CP.

One of the most variables of concern when pigs are fed LP diets is the fatter carcass compared with HP diets [9, 10], which may be partially due to more dietary energy being available for fat deposition in LP diets [4]. The adoption of the net energy (NE) system when formulating LP diets for pigs has been suggested by several authors in the last few years as means to still achieve acceptable performance, carcass characteristic and meat quality [4, 11, 12].

To give a better understanding of LP diets and provide a reference for further study, this paper reviews the advantages of LP diets in reducing feed costs and nitrogen excretion, and promoting gut health. The influence on growth performance and the application of the NE system in LP diets have also been highlighted. Finally, a summary of the published recommendations for dietary standardized ileal digestible (SID) EAA and NE in LP diets are provided.

## The advantages of LP diets

### Saving protein ingredients and reducing feed costs of pigs

**Fig. 1:**
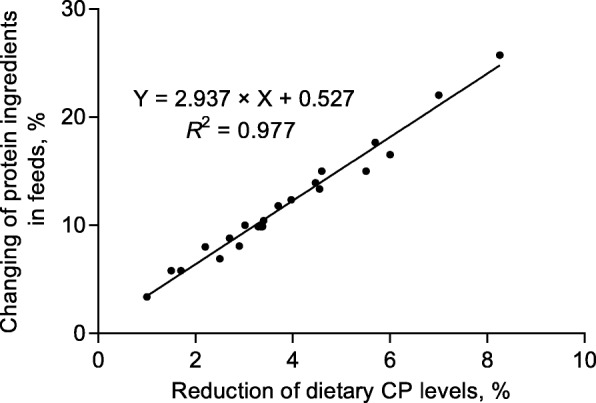
Linear relationship between the reduced percentage of dietary protein ingredients and CP reduction levels for pigs. The data of regression analysis summarized from 10 published research articles evaluating the effect of LP diet on pig performance [1, 5, 19, 38, 77, 130–134]

**Fig. 2:**
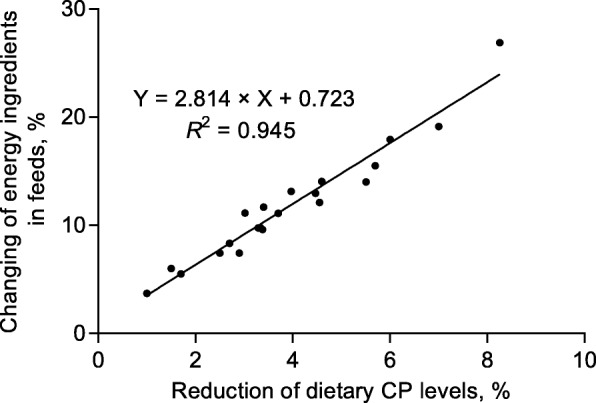
Linear relationship between the increased percentage of dietary energy ingredients and CP reduction levels for pigs. The data of regression analysis summarized from 10 published research articles evaluating the effect of LP diet on pig performance [1, 5, 19, 38, 77, 130–134]

The shortage of high quality protein sources is a worldwide problem, especially in China. Globally, China is the largest soybean importer since 2002 [13]. In 2016, China’s soybean imports were 8,391 million tons and accounted for more than 26% of the worldwide production; therefore, reducing the dietary protein content could effectively reduce pressure on protein ingredient supplies. Summarizing recent studies evaluating LP diets fed to pigs, we can conclude that every 10 g/kg reduction of dietary CP resulted in a 3% reduction in protein ingredient inclusion (Fig. 1). Interestingly, the energy source is increased by almost 3%, equal to the reduction in protein source of every 10 g/kg reduction of dietary CP (Fig. 2). Thus, the application of LP diets can also be considered a cost-effective alternative feeding strategy due to higher price of protein ingredients versus energy sources. The SBM is the most widely fed protein source in pig diets, with a high and consistent product quality, has more than doubled over the last 7 years due to less acreage used to grow soybeans [14]. For instance, the price ratio of SBM to energy feeds (corn and wheat are the most widely feed energy sources) is more than 150% in China. Other protein ingredients like cottonseed meal, rapeseed meal and distillers dried grains with soluble (DDGS) are also more expensive than energy feeds [15]. Therefore, considering less protein ingredient inclusion and greater energy source inclusion and the cost of additional supplemented CAA in LP diets, every 10 g/kg of reduction of CP could decrease feed cost by about 1.50% in China [16]. The exact savings of LP diets to pig production are influenced by other factors, including the fluctuations in the market price of feedstuffs and CAA, feed cost per kilogram of BW gain and the value of products obtained for a given performance, and the reduced cost of environmental protection.

### Nitrogen emission

**Fig. 3:**
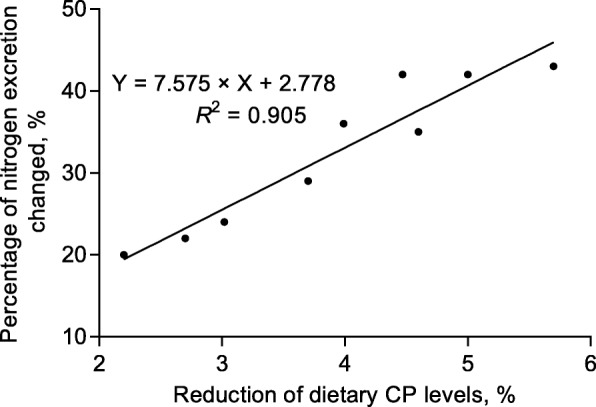
Linear relationship between the reduced percentage of nitrogen excretion and dietary CP reduction levels for pigs. The data of regression analysis summarized from 7 published research articles evaluating the effect of LP diet on nitrogen excretion [1, 38, 77, 130–132, 134]

Nitrogen excretion is the major criticism in modern pig production because of the negative impact on the environment, such as manure ammonia (NH_3_) contribution to acidification and eutrophication of sensitive ecosystems and odor emissions [17]. The concentration of CP in traditional CSBM diets is elevated in order to satisfy the requirement of lysine because lysine is the first limiting AA in CSBM based diets. Consequently, the overabundance of other AA is broken down into nitrogen and the excess nitrogen excreted in the urine as urea. One effective approach to decreasing the nitrogen emission is to reduce the content of dietary CP, supplementing with CAA, to come close to matching the pig’s ideal protein pattern. Based on an assumed ideal protein pattern, the LP diets improved the utilization efficiency of nitrogen without affecting the digestibility and retention of nitrogen [10]. Most studies indicated that a reduction of dietary CP more than 20 g/kg could effectively decrease the nitrogen emission. The effect of dietary CP reduced levels on nitrogen excretion of pigs are represented in Fig. 3. Every 10 g/kg reduction of dietary CP can decrease ammonia emission from feces and urine by 8% to 10%. In addition, lower dietary CP level resulted in reduced water intake, along with decreased urea nitrogen excretion in urine [5, 18]. Serum or plasma urea nitrogen (SUN or PUN), the main and ultimate nitrogenous product of protein catabolism, has also been detected at lower concentrations in pigs fed with LP diets compared with the normal protein diets [19]. Therefore, these two factors can be used as easily determinable indicators of reduced nitrogen excretion [20].

Odor emissions from pig production units can be a nuisance in the surrounding area. Often the production of odorants is a consequence of anaerobic fermentation of undigested proteins in animal manure [17]. In this respect, the reduction of dietary CP could decrease odor emissions. Hayes et al. [21] found odor emission rates were significantly reduced by 31% and 33% for the 160 and 130 g/kg CP diets in comparison to the 190 g/kg CP diet, and odor emission from pigs fed 190 g/kg CP diet was similar to the CP level of 220 g/kg. Alternatively, Leek et al. [22] studied a similar range of dietary CP levels (130, 160, 190 and 210 g/kg) and reported that the odor emission rate was lowest at 160 g/kg CP but increased as the CP concentration was reduced to 130 g/kg. This contradictory response in odor emission may be related to a higher level of sulfur-containing AA (supplemented crystalline *DL*-methionine) in 130 g/kg CP diets because the fermentation products of sulfur-containing AA including H_2_S, dimethylsulphide, dimethyldisulphide and dimethyltrisulphide have been correlated with odor concentration [23].

### Gut health

Many studies have reported that LP diets effectively lowered the incidence of post-weaning diarrhea (PWD), maintain gut health, and had distinct influence on intestinal morphology and microbiota.

#### Lowering incidence of post-weaning diarrhea of piglets

In weaning period, the feed is transited from liquid (milk) to solid, accompanied with changes of psychological, environment, morphological and microbial in the gastro-intestinal tract (GIT) of young pigs [24]. These changes are often associated with a high incidence of PWD in this period. In-feed antibiotics have been extensively used as an effective preventative measures for PWD [25]; however, the appearance of antimicrobial resistance (AMR) caused a global concern about the negative effects of sub-therapeutic use of antibiotics. As a result, a ban on the use of in-feed antibiotics was introduced in 2006 in the Europe, and the World Health Organization (WHO) released its Global Action Plan on AMR in 2015. Accordingly, alternative nutritional strategies to control the PWD are useful options and also will not cause security concern, such as reducing dietary CP of weanling piglets.

The fermentation of undigested protein and AA by the intestinal microbiota are important factors contributing to PWD [26]. Numerous metabolites are produced from protein fermentation in the hindgut, such as short- or branched-chain fatty acids (SCFAs, BCFAs) [27], sulfur-containing bacterial metabolites (methanethiol, H_2_S), aromatic compounds (phenolic, indolic compounds), polyamines, and ammonia [28]. Some of the metabolites are beneficial to the host; for example, SCFAs may be used as an energy source by the host or regulate cell proliferation and differentiation and BCFAs have been shown to regulate electrolyte absorption and secretion [29, 30]. However, the relative proportions of fatty acids that come from protein fermentation are present at much lower concentrations and their production is mainly regulated by available concentrations of carbohydrates [31]. The ammonia, polyamine, phenol and indole products of fermentation represent potential toxins to host health, such as cytopathic effects on epithelia cells, or inhibiting mitochondrial oxygen as metabolic troublemakers towards colonocyte energy metabolism [32]. Moreover, HP diets have higher acid-binding capacity and increase pH of the GIT to nearly neutral conditions, which provide a favorable environment for the proliferation of pathogenic bacteria, such as *Bacteroides* and *Clostridium* species, thereby increasing PWD incidence [33]. Thus, reducing the amount of protein reaching the large intestine by selecting highly digestible protein ingredients or lowering the dietary protein level may help to alleviate PWD. Although a full description of protein sources is beyond the scope of this article, animal protein exhibits a superior feeding value than plant protein partly due to higher digestibility [34]; therefore fishmeal and whey powder often used in young pig diets.

The CP level of typical CSBM diets for weaned piglets in the nursery phase (i.e. from 7 to 20 kg body weight) commonly varies from 21% to 25% according to the hygiene situation of pig farms or production goals [35]. Many studies have investigated the extent of dietary CP reduction needed to effectively lower PWD. Yue and Qiao [6] reduced the dietary CP level from 23.1% to 18.9% of supplementing with FCAA and reported lower PWD and a simultaneous improvement in fecal consistency. A further reduction to 17.2% decreased growth performance despite addition of *L*-isoleucine, *L*-valine, *L*-histidine, and *L*-phenylalanine to achieve an ideal AA pattern. Therefore, it was suggested that reduced dietary CP for piglets should be no more than 4 percentage units below NRC (1998) [3] or 2 percentage units below NRC (2012) [8] recommendations because other non-essential amino acids (NEAA) may become limiting with further reduction resulting in poor performance [1, 5]. However, a study of long-term feeding of LP diets for weanling piglets indicated that a reduction of CP levels from 24.3% to 17.3% supplemented with EAA to conform to an ideal AA pattern can reduce the PUN and NH_3_-N, resulting in lower PWD of weanling piglets over the whole period without compromising production [36]. Similar results were reported in a subsequent study using pigs infected with an enterotoxigenic strain of *Escherichia coli* (*E. coli*) [37]. Many other studies also showed a similar trend in the incidence of PWD when feeding LP diets (not less than 17%) to weanling piglets [38, 39].

#### Intestinal morphology

Evidence suggests that LP diets have no significant influence on the intestinal and mucosal weight [40]. Pigs fed LP diets exhibited decreased crypt depth in sections of the small intestine and a tendency for greater villus height to crypt depth ratio, which implied that LP diets could effectively improve the digestion and absorption of the small intestine [41]. Deeper crypts and notable villus atrophy were observed 3 d after enterotoxigenic *E. coli* (ETEC) challenge in the ileum of weaned piglets fed a 22.5% CP diet compared with those fed a 17.6% CP diet [42]. This result further confirmed that LP diets could alleviate alterations in intestinal morphology induced by pathogenic bacteria and maintain the normal digestion and absorption capacity of intestinal cells. However, further reduction by more than 4% resulted in a significant reduction in villous height in both duodenum and jejunum even though diets were supplemented with *L*-isoleucine, *L*-valine, *L*-histidine and *L*-phenylalanine [6, 7]. The reduction of villous height in pigs fed LP diets are likely related to less protein to maintain the architecture of intestinal epithelium. In general, reducing the dietary CP level within a suitable range not more than 3 percentage units will not modify the intestinal morphology integrity [40].

#### Intestinal microbiota

The mammalian intestine is colonized with many thousands of microbiota strains with the total number of microbial cells exceeding 10^14^ [43]. Intestinal microbiota play an important role in host physiology and metabolism [44]. Undigested dietary protein fermentation is possibly associated with an enhanced proliferation of protein-fermenting bacteria [45]. Therefore, the source, quality and level of dietary protein may have an influence on microbial communities. Many studies reported that dietary CP level rather than its source has more significant impacts on the composition of the intestinal microbiota [24]. The influence of dietary CP levels on the intestinal microbiota communities has been more widely studied in weaned piglets, because the structure of bacterial composition of growing or finishing pigs remains relatively stable. In a study with newly weaned piglets, reduced dietary CP decreased counts of *Clostridium leptum*, but had no effect on total bacteria, *Lactobacilli*, *Enterobacteria* and *Bacteroides* [46]. A culture-independent method using denaturing gradient gel electrophoresis (DGGE) indicated that the numbers of *Firmicutes* and *Clostridium cluster* IV were lower in pigs fed 14% dietary CP than in 20% dietary CP with minimal impact on other bacteria populations [47]. Principally, it seemed that the dietary CP level influences the *Clostridium* genus. Opapeju et al. [42] reported that in young pigs challenged with ETEC, reducing dietary CP can significantly lower the amount of ETEC in both ileum and colon. However, many other studies reported that changes in dietary CP content had no significant influence on the bacterial communities in all sections of the intestine under normal physiological conditions, because the microbiota can adapt to a certain extent following changes in dietary CP level [48]. Results of studies evaluating the effect of dietary CP level on the composition of microbiota are inconsistent. However, many of these studies were performed using the traditional culture-dependent or low throughput culture-independent methods and these methods are limited to detection of bacteria that can be cultured in vitro or are abundant in vivo.

With the application of high-throughput sequencing, 16S rRNA gene sequencing has been widely used in biological studies and may help in developing a comprehensive understanding of the influence of dietary CP level on variation in intestinal bacteria. In a long-term feeding experiment (100 d), pyrosequencing of the V1-V3 region of the 16S rRNA gene showed a 3% reduction in dietary CP decreased the relative abundance of *Lactobacillus* in the cecum and *Streptococcus* in the colon of growing pigs compared with a HP diet [49]. Moreover, the ileum and colon microbiota of finishing pigs were also influenced by dietary CP levels, where a moderate reduction of dietary CP concentration (no more than 3%) improved the bacterial community structure in both the ileum and colon of finishing pigs; specifically increases in the proportion of Peptostreptococcaceae which are helpful in maintaining gut homeostasis [7].

Conventional wisdom suggested that adult mammals have a stable intestinal microbiota population that is difficult to influence by dietary strategies in comparison to weaned piglets harboring unstable microbiota populations [50]. Additionally, previous studies focused on the changes of hindgut microbiota and neglected the effect of bacteria in the small intestine [51]. With the application of high-throughput sequencing, the microbial communities of different physiological stages and gut locations have provided evidence that the intestinal microbiota population may be changed by different dietary CP contents.

#### Immune response

Soybean contains many anti-nutritional factors including trypsin inhibitor, agglutinins, antigenic proteins, isoflavones and alkaloids which impede the digestion and absorption of nutrients [52]. Furthermore, undigested antigen proteins in soybeans can enter the lymph and blood through the gaps between intestinal epithelial cells and stimulate hypersensitivity for weanling piglets [53]. Therefore, lowering the CP level as a nutritional strategy to decrease the soybean content of diets could be in part, ameliorate immune stress of piglets at weaning [54].

Growing evidence has demonstrated that supplementing LP diets with free AA can have various biological effects besides use for protein synthesis. For example, dietary supplementation with CAA modulates gene expression, reduces excessive body fat, enhances growth of intestine and skeletal muscle and contributes to immunologic defense and minimizing gut disorder [55–57]. Particularly under stress conditions, supplementing with CAA in LP diets can modify immune status and improve the resistance to subclinical and clinical diseases [58].

##### Threonine

A sufficient intake of dietary threonine is particularly important for mucosal mucin synthesis and maintaining integrity of the gut barrier [59]. Wang et al. [57] reported that an increase in true ileal digestible (TID) threonine from 0.37% to 0.89% with 16% CP in weanling pigs can significantly improve the concentrations of ileal acidomucins, duodenal sulfomucins and the total amounts of mucins in the duodenum. However, a further increase to 1.11% resulted in downregulation of intestinal mucin expression and derangement of the intestinal mucosal epithelial barrier. Serum IgG concentrations also increased in response to increased intake of TID threonine where 0.85% threonine intake was the optimum level [59]. In addition, the threonine required to maximize the immune response is greater than for maximum weight gain and increased threonine levels in LP diet increased plasma antibody concentration [60].

##### Sulfur amino acids

Sulfur amino acids (SAA) are important for the immune system; methionine provides the methyl group for the synthesis of spermidine and spermine which is important for the proliferation and differentiation of lymphocytes [61]. Cysteine is the precursor of glutathione (GSH), homocysteine (Hcy) and taurine which play crucial roles in the response to immunological challenges. A deficiency of cysteine suppresses epithelial growth, impairs the proliferation of lymphocytes, reduces the production of CD4 cells and IFNγ, and diminishes T-cell activity [62]. Under immune system stimulation, additional SAA intake had an obligation to maintain whole body protein synthesis due to increased immune protein synthesis [63].

##### Tryptophan

Tryptophan plays a role in immune responses via products of its catabolism, such as serotonin, melatonin and N-acetylserotonin which can inhibit the production of superoxide and TNFα, eliminate free radicals, and modulate inducible nitric oxide (NO) synthesis [56]. Additionally, available evidence indicates that tryptophan catabolism may produce a local immunosuppressive effect to control T-cell homeostasis during inflammation [64]. Trevisi et al. [65] reported that supplementation of 0.1% *L*-tryptophan to a standard weaning diet (CP 19%, analyzed tryptophan: lysine = 0.20) allowed piglets with a greater susceptibility to ETEC K88 and maintain an adequate body growth rate. In growing pigs fed LP diets, dietary SID tryptophan to lysine ratio of at least 22% maximized production potential and increased resistance to inflammation and immune response under commercial conditions [66]. Similar results were reported in a recent study from the weaning to finishing phase conducted under different sanitary conditions [58].

##### Branched-chain amino acids

Branched-chain amino acids (BCAA) provide the carbon skeletons for endogenous synthesis of glutamine, which is an abundant AA in plasma, skeletal muscle, fetal fluids and milk and is a major energy substrate for immune cells [67]. The BCAA can also be used as the precursors for the synthesis of new immune cells, effector molecules, and protective molecules [68]. A recent study reported that a protein restricted diet (CP 17%, analyzed leucine, valine, and isoleucine to lysine ratio was 0.87, 0.51, and 0.41, respectively) supplemented with BCAA (0.07% *L*-leucine, 0.27% *L*-valine, and 0.19% *L*-isoleucine) could improve intestinal immune defense functions by protecting villous morphology and increasing intestinal immunoglobulins levels in weaned piglets [69].

##### Lysine and arginine

In general, a lysine deficiency would not directly affect the host immune response, but would limit the synthesis of proteins (including cytokines) and the proliferation of lymphocytes [70]. Further, because lysine shares the same transport systems with arginine, the content of dietary available lysine can modulate the metabolism of arginine [71]. Arginine is a nutritionally EAA for piglets due to insufficient endogenous synthesis and it has been reported that dietary arginine supplementation could enhance immune response of weaned pigs [72]. N-carbamylglutamate (NCG) has been demonstrated to increase endogenous synthesis of arginine by activating intestinal pyrroline-5-carboxylate synthase and carbamylphosphate synthase-1 [73]. It is widely used as a feed additive instead of *L*-arginine-HCl because of concerns over the short biological half-life of arginine and the arginine/lysine antagonism [74]. Recent studies have demonstrated that NCG supplementation has some beneficial effects on intestinal mucosal immunity associated with stimulated lymphocyte proliferation and cytokine synthesis [75].

### Inclusion of fermentable carbohydrates in LP diets

Recently, the inclusion of moderately fermentable carbohydrates seems to be the most promising approach to maximize the efficacy of LP diets and has been presented in considerable detail by other reviews [17, 24, 76] and won’t be discussed in detail here. However, from these reviews we can conclude that the inclusion of fermentable carbohydrates into the LP diet may provide the following advantages in pig production: 1) divert nitrogen excretion from urine to feces; 2) increase SCFAs production while reducing the slurry pH; 3) the carbohydrates would be preferential substrates for microbial fermentation over protein. Combined with the reduction in excretion of nitrogen, emission of NH_3,_ incidence of diarrhea and production of odorants would also be reduced; all of which are beneficial to piglet growth and the environment.

## Influence of dietary CP level on pig production performance

Low-protein diets have the advantage of reducing feed cost and nitrogen excretion, and amelioration of the diarrhea rate of weaning piglets. However, pig production performance should not be compromised when feeding with LP diets. Therefore, the influence of dietary CP level on growth performance, carcass characteristic and meat quality are included as an important criterion to justify LP diets.

### Growth performance

Growth performance, including ADG, ADFI and F:G is the first factor to evaluate LP diets and also an indicator that is relatively simple to determine. It is well accepted that dietary CP reduction within 3% of the NRC (1998) [3] and supplemented with FCAA can result in similar growth performance in growing-finishing pigs as those of the control diets [77].

However, conflicting results are reported on the influence of a further reduction in dietary CP level on growth performance by more than 3% (Table 1). Retarded growth performance was reported when the CP level was reduced by more than 3% with only FCAA supplemented in diets [6]. Roux et al. [78] reported that a reduction of CP by 4.8% along with FCAA supplementation resulted in significantly decreased growth performance compared with pigs fed HP diets. Whereas, the reduced performance was alleviated with valine and isoleucine supplementation implying that BCAA may be the next-limiting AA in LP diets. Similar results were reported by Powell et al. [79]. Supplementing BCAA to LP diets can increase growth performance by increasing the feed intake and skeletal muscle growth in piglets [80]. Zhang et al. [19] also showed that dietary BCAA content was the limiting factor affecting the growth performance of fattening pigs with 4.5% reduction in dietary CP content.

**Table 1:** The variation in performance of pigs fed diets with CP reduced by more than 3%

BW, kg	Dietary CP level^a^, %		Additional supplemented AA^b^	Performance changed^c^			References
	Control diet	LP diet		ADG, g/d	ADFI, kg/d	F:G, kg/kg	
24~ 40	24.1	19.3	Valine	−38	−0.24	−0.12	Morales et al. [81]
		18.1	Valine, leucine, isoleucine, histidine, phenylalanine	−33	− 0.06	−0.01	
		17.3	Valine, leucine, isoleucine, histidine, phenylalanine, glycine	−13	−0.04	−0.03	
7~ 11	23.1	18.9	–	14^d^	0.02	−0.01	Yue and Qiao [6]
		17.2	Valine, isoleucine, histidine, phenylalanine	57^d^	0.50	− 0.14^d^	
6~ 13	22.8	17.4	Isoleucine	121^d^	0.11^d^	−0.30^d^	Nyachoti et al. [5]
50~ 80	19.5	12.0	Valine, leucine, isoleucine, histidine	111^d^	0.07	0.25^d^	Atakora et al. [135]
10~ 20	19.4	12.7	Valine, histidine, isoleucine, leucine, phenylalanine	70^a^	−0.01	− 0.35^d^	Gloaguen et al. [1]
20~ 50	18.2	13.3	–	44^d^	0.03	−0.09	Powell et al. [79]
		13.4	Valine, isoleucine	27	− 0.02	−0.12	
		!	Glycine, arginine	64^d^	0.08	−0.10	
		!	Valine, isoleucine, glycine, arginine	13	0.04	0.02	
		!	Valine, isoleucine, glycine, glutamic acid	8	0.03	0.02	
20~ 45	18.2	13.3	–	56^d^	−0.01	−0.26^d^	Roux et al. [78]
		13.4	Valine, isoleucine	30	− 0.12^d^	−0.26^d^	
37~ 61	16.1	10.1	Valine, isoleucine, phenylalanine	171^d^	0.01	−0.44^d^	Guay et al. [40]
		7.8	Valine, isoleucine, phenylalanine, histidine, leucine, arginine	313^d^	0.06	− 0.97^d^	
75~ 95	14.7	10.1	Valine, isoleucine, alanine	170	0.14	−0.63	Zhang et al. [19]
		10.2	Valine, isoleucine, leucine	90	0.14	− 0.18	

^a^Analyzed values; “!” means data were not provided in the paper

^b^Additional supplemented AA in LP diets except FCAA; “-” means no other CAA supplemented except FCAA

^c^ADG = ADG (control diet)- ADG (LP diet); ADFI = ADFI (control diet)- ADFI (LP diet); F:G = F:G (control diet)- F:G (LP diet)

^d^ Values were significant different between LP diet and control diet

Further reduction is more than 6% CP level would be possible when supplemented with all EAA without affecting pig growth performance, which implies that NEAA does not seem to be limited in very low-protein (VLP) diets for growing pigs [81]. However, Guay et al. [40] reported poor performance in growing pigs fed diets with 8.3% CP reduction even though all the EAA were added to a same level as the control group (16.1% dietary CP). This suggests that insufficient NEAA or total nitrogen may be the reason for the poor growth performance [82]. In addition, some AA that are typically considered as NEAA may become essential when the dietary CP is below a certain level in the LP diet [83]. The conditionally EAA arginine and cysteine would likely be the first-limiting AA and dependent on de novo synthesis to optimize growth in VLP diets while the EAA were satisfied [79]. Nevertheless, Gloaguen et al. [1] showed that young pigs fed a 12.7% CP diet, supplemented with sufficient amount of EAA and NEAA, had reduced growth performance compared with pigs fed a 19.7% CP diet. The poor performance in pigs fed VLP diet may be due to a deficiency in intact protein or excess of free AA. Inclusion of protein-bound AA was reported to be a more effective strategy to maintain nitrogen retention and whole body protein homeostasis than free AA [40]. Moreover, the content of di- and tri-peptides hydrolyzed from intact protein was reported to be positively correlated with the activities of digestive enzymes [84]. The more rapid absorption rates of free AA may induce excessive oxidation of AA contributing to the decreased body protein deposition and poor growth performance [85].

Discrepancies of growth performance caused by LP diets between the above studies may be due to variations of CP levels designed for the control diets, types of feed ingredients, feeding pattern (restricted vs. ad libitum feed intake) and experimental conditions. In general, the first four limiting AA should be supplemented when the CP reduction is below 3%, additional supplementation with BCAA could maintain similar growth performance compared with control group with up to a 6% reduction. Further reduction (more than 6% reduction of CP content) will require inclusion of dietary nitrogen or sufficient NEAA to ameliorate the poor growth performance.

### Carcass characteristic

It’s debatable whether LP diets supplemented with CAA affect carcass characteristics. Dressing percentage is the ratio of carcass weight to pre-slaughter live weight and is not affected by reducing dietary CP level [9, 86, 87]. However, increased back-fat thickness and reduced loin muscle area at slaughter were consistently reported in LP diets studies [4, 88, 89], in fattening pigs as well as growing pigs [10, 90]. Three reasons may be responsible for this observation: 1) more energy is required for excretion of excess AA in the HP diet. In contrast, LP diet are closer to the ideal protein ratio which would reduce energy needed for N excretion leaving more energy to be deposited in adipose tissue [91]; 2) the relative weight of liver, intestine, kidney and pancreas increases in HP diets which increases maintenance energy needs [81, 88]; 3) the relatively greater proportion of cereal grains in a LP diet results in a high content of available starch which is more efficient for fat deposition than AA [92]. To alleviate the negative effects on carcass characteristic of LP diets, many researches had been conducted. Supplementation with some functional AA have shown potential to reduce excessive body fat (e.g. leucine, arginine, glutamine, glutamate, and proline) [93]. Leucine, a BCAA, is reported to be an effectively AA to regulate protein synthesis through the mammalian target of rapamycin (mTOR)-dependent process [94]. Zhang et al. [19] showed that the supplementation of 0.40% *L*-leucine to a LP diet (CP 10%, SID leucine: lysine = 1.05) increased tissue protein synthesis and improve the carcass characteristic of finishing pigs more than an alanine-supplemented diet when compared with the control diet (14.5%). Some studies have demonstrated that dietary arginine, a semi-essential AA, may reduce fat deposition and stimulate protein synthesis by regulating metabolism of energy substrates through production of NO [95]. However, an imbalance of energy to nitrogen may be the primary reason for the fatter carcass in pigs fed LP diets. The NE system (in comparison with digestible energy (DE) or metabolizable energy (ME) systems) has been confirmed to be superior for controlling carcass adiposity and will be detailed in the following sections.

### Meat quality

Meat quality is primarily assessed by the following sensory traits: color, pH, marbling (intramuscular fat (IMF) content), tenderness (shearing force), water holding capacity (WHC; drip loss, purge loss, cooking loss), and juiciness [96]. Most studies reported no significant difference in pH_24 h_ and WHC in pigs fed different dietary CP [97, 98]. Some variables (L^*^, a^*^, b^*^, C^*^ and H^0^) of meat color increased when dietary CP was restricted [87, 97]. Nevertheless, other studies reported no differences were observed in L^*^ between treatments [98, 99]. Zanardi et al. [100] suggested that human perception of pork color is strictly linked with lightness and hue angle which are typically affected by pH and WHC, while redness and chroma appear to be less important. Recent studies suggested a tendency towards greater fat deposition in pigs fed with LP diets from 40 to 115 kg BW, concomitantly pork that was more tender and juicy was reported [101]. Suárez-Belloch et al. [87] also observed that dietary CP restriction during the growing period improved some traits related to meat quality, such as decreasing the hardness and tending to increase the IMF content which was desirable for heavy pigs intended for dry-cured products. In the study of Teye et al. [102], marbling fat in longissimus dorsi muscle was 2.9% in pigs fed LP diets compared with 1.7% in pigs fed HP diets and the backfat thickness was similar between LP and HP-fed pigs. Scores of tenderness and juiciness were also markedly increased in pigs fed LP diets by 0.6 units and 0.5 units, respectively. A higher percentage of oleic acid and a lower percentage of linoleic acid in both longissimus and semimembranosus muscles were determined in LP diets which suggest an improved eating quality [101]. These sensory traits of meat quality are mainly dependent on two related biological processes including muscle growth and deposition of fat [103].

Muscle growth reflects protein accretion which is a balance between the rates of protein synthesis and degradation [104]. The mammalian target of rapamycin complex 1 (mTORC1) pathway plays a decisive role in controlling protein synthesis [105]. The mTORC1 pathway includes the following components, Raptor: an adaptor to recruit substrates to the mTOR protein; 4E-BP1 and S6 K1: two of the most characterized substrates for the mTORC1 pathway, activation of the pathway promotes protein translation and increases cell growth [106]. Accumulating data demonstrates there is a positive relationship between dietary CP level and mTOR signaling of muscle protein synthesis in pigs [107]. At present, the process of protein degradation as influenced by protein levels has received much less attention. Zhou et al. [86] observed the mRNA abundance of FOXO, MAFbx and MuRF1 that included in the ubiquitin-proteasome pathway (UPP) was not influenced when the CP level reduced from 14% to 10% in growing pigs. The UPP is the primary intracellular system for protein degradation in skeletal muscle, which implied that the proteolysis of skeletal muscle was not affected by the dietary protein restriction. Moreover, dietary BCAA supplementation enhanced muscle mass due to a greater increase in protein synthesis than protein degradation [104].

Fat is an important contributor to various aspects of meat quality [103]. However, fat is also considered an unhealthy constituent for many consumers [108]. Therefore, manipulation of the fatty acid composition of muscle and fatty tissues has achieved great interest in the last decades. Intramuscular fat has been reduced to below 1% of muscle weight in modern pigs through the genetic selection which has simultaneously harmed the sensory trait of pork because of a direct relationship between IMF and the formation of tenderness, juiciness, marbling, and the flavor of cooked meat [98]. Therefore, consumers are becoming increasingly interested in meat containing high concentration of IMF and polyunsaturated fatty acids (PUFA) [109], as seen that pigs fed with LP diets dramatically enhance the content of IMF in growing or finishing phases [12, 101]. Growing-finishing pigs fed a LP diet showed a high content of IMF in the longissimus thoracis muscle. In addition, supplementation with antioxidants like essential oil and benzoic acid further decreased the degradation of lipids in muscles [110]. A study conducted in two different genotypes further suggested that dietary CP regulated lipid anabolism and catabolism via modulation of the mRNA levels of key regulatory enzymes and fatty acid transport proteins in different muscle tissues [103].

Taken together, although an appropriate reduction of CP level would increase the IMF content and tenderness score, consequently ameliorating the meat quality concern, the reduction in protein synthesis and leanness is out of our expectation. Therefore, further research on the underlying mechanisms of how LP diets influence the meat quality need to be conducted.

### The effects of LP diets on nutrient digestibility

In digestibility studies, digestibility of different nutrients has generally been evaluated separately, because the relationship between the digestibility of these nutrients is complicated making it hard to determine the interactive effects. However, some attempts had been made to reveal the complex interaction.

It has been generally assumed that AA and phosphorus (P) digestion and absorption were independent in swine; for example, dietary CP concentration was not considered in the studies of P digestibility. However, AA and P are two key regulators and components to the skeletal muscle growth and gene expression of the active transporter of P in the small intestine is limited by CP restriction [111]. Moreover, Xue et al. [112] indicated that the content of ileal digested P decreased in the LP diet compared with the HP diet of growing pigs, which indicate a limiting effect of dietary CP level on ileal P digestion. A possible reason may be that limited protein intake impaired the active transportation system of P.

An earlier study reported higher ileal digestibility of fat and saturated fatty acids 14:0 and 18:0 with increasing dietary CP [113]. However, other studies reported a lower digestibility of crude fat with increasing dietary CP concluding that free fatty acids can bind with undigested protein to form micelles that are unavailable for absorption [114]. In addition, the relative proportion of endogenous fat excretion into the intestine increased in HP diets may affect the apparent digestibility of fat.

There have been no studies specifically evaluating the effect of LP diets on fiber digestibility; whereas many studies have demonstrated that fiber and LP diets have synergetic effects on gut health [24]. Therefore, the influence of LP diets on the digestion of fiber should be determined.

## The nutrient balance of LP diets

### The NE requirement and the ratio of NE to lysine for LP diets

The most concerning challenge of LP diets supplemented with CAA on pig production is the increase in back-fat at slaughter [4, 9] and may be partially due to more dietary energy being available for fat deposition. The reduction in deamination of excess AA and nitrogen and thus, less heat production, reduces overall energy expenditure [11, 91]. The DE or ME system over-estimates the available energy of protein and simultaneously underestimates starch energy availability. The proportional value of NE/ME for protein is 60% compared with 80% for starch [115]. Chen et al. [116] reported that NE is the only system where energy requirements of the animal and available energy of all feed types can be expressed on the same basis. Therefore, the NE system may be more suitable for LP diets and would help to inhibit excessive carcass fat deposition. Le Bellego et al. [117] confirmed the superiority of using the NE system in diets with variable CP contents by measuring the growth performance and carcass adiposity in comparison with DE or ME systems. However, accurate measurement of the NE value of feedstuff was still be a part of the problem that hampers the application of the NE system, because the NE is harder and more difficult to determine than DE and ME for pigs [116].

Yi [118] conducted eight experiments to investigate the requirements of NE for growing and finishing pigs fed LP diets supplemented with CAA. The first four experiments indicated that the optimal NE level was 2.36 Mcal/kg and 2.40 Mcal/kg for growing and finishing pigs calculated using both the broken-line model and quadratic regression analysis with maximum performance and carcass characteristics, respectively. The following two experiments suggested the best ratio of lysine to NE was 4.70 g/Mcal and 3.50 g/Mcal when dietary CP was reduced by four percentage units for growing and finishing pigs, respectively. Recommendation requirements from the six previous experiments have been validated in commercial conditions in two subsequent experiments. To the best of our knowledge, this study is the only comprehensive evaluation of the optimal NE levels in LP diets for pigs in different growing phases under commercial conditions.

### The AA balance requirements of SID AA in LP diets

To eliminate the invalid fermentation of AA in hindgut and higher correlation was observed between both daily gain and feed efficiency with ileal AA digestibility than fecal values, ileal digestibility is more suitable than total tract digestibility for AA bioavailability. Moreover, SID AA is widely used because the contribution of basal endogenous losses are quantified and more likely to be additive in mixed diets, thus, it is more accurate than apparent ileal digestible (AID) values [119]. The NRC (2012) [8] has published the SID AA requirements in different growing phases of pigs with ad libitum feed access. However, the recommended AA needs were determined using a prediction model and limited empirical studies have been conducted to validate the values. In addition, the estimates are based on traditional dietary CP concentrations that may be inconsistent with CP levels common in LP diets. Therefore, a precise measurement of the appropriate SID AA requirements when pigs are fed LP diets could maximize the advantages of LP diets and simultaneously optimize the cost of supplemental CAA.

The SID AA requirements of pigs are expressed as the AA to lysine ratio and estimated using dose-response studies representing the sum of those for body maintenance functions and protein retention [8]. When determining the AA requirements (except lysine), lysine should be at a suboptimal level (second limiting) after the tested AA which should be tested with at least four different gradient levels [120]. Performance, carcass traits, serum AA concentrations and SUN are used as the response criteria for growing pigs [121] and the ‘best’ criteria should base on the specific experiment. For instance, growth performance is better for growing period, carcass traits are better for fattening period, serum AA can be used to validate estimates and SUN has been used as a rapid response criterion [122]. In dose-response studies, the choice of statistical method used to interpret the data is critical. The most widely used models include the linear-broken line analysis and the quadratic model each with its advantages and disadvantages, which have been completely summarized by Robbins et al. [123].

The order of the first four limiting EAA is lysine, threonine, tryptophan and SAA in CSBM based LP diets according to the extent of additional supplemented CAA compared with the HP diet. This order is a little different from the NRC (2012) [8] recommendations with a sequence of lysine, SAA, threonine and tryptophan [124, 125]. Moreover, recent studies showed that besides these four EAA, valine can also contribute to the improvement of growth performance as the fifth limiting AA [122].

Many of the studies on requirements for these five EAA in pigs fed LP diets across the entire growth phases for commercial pigs have been completed by our group [124–129]. Appropriate requirements of CP, NE, and SID AA in growing pigs fed with LP diets based on these studies determined in China are summarized in Table 2. The recommended requirements of SID AA determined in these studies were slightly higher than corresponding NRC (2012) [8] recommendations, indicating that more nitrogen from CAA is utilized for the synthesis of deficient NEAA than when pigs are fed normal protein diets. In addition, these studies have been primarily conducted under commercial conditions and are more applicable for pig production than the NRC (2012) [8] requirements determined under laboratory conditions or model derived. Moreover, our unpublished data had been confirmed the recommended requirements of Table 2 were applicable in commercial pig farms in China.

**Table 2:** Dietary protein, NE and SID EAA requirements of growing pigs^a^

Item	Body weight range, kg			
	7-20	20-50	50-80	80-110
Crude protein, %	18	15	13	12
NE content^b^, kcal/kg	2450	2360	2360	2400
Amino acids, standardized ileal digestible basis, %				
Lysine	1.30	1.01	0.86	0.75
Threonine	0.84	0.65	0.54	0.49
Tryptophan	0.26	0.18	0.15	0.13
Methionine + Cysteine	0.75	0.58	0.50	0.43
Valine	0.78	0.63	0.56	0.51
Ratio of SID AA to SID lysine				
Threonine/Lysine	0.65	0.64	0.63	0.65
Tryptophan/Lysine	0.20	0.18	0.17	0.17
(Methionine+ Cysteine)/Lysine	0.58	0.57	0.58	0.57
Valine/Lysine	0.60	0.62	0.65	0.68

^a^The recommended requirements summarized from the academic dissertation of our research group determined in China [118, 124–129]

^b^The NE values of feed gradients are estimated based on Noblet’s equations [115]

## Conclusion

The application of LP diets is a tool in pig production not only to reduce feed costs and nitrogen excretion but also to effectively improve gut health in an era of increasing antibiotic use limitations. Reducing CP level by three to four percentages and supplementing with crystalline lysine, threonine, tryptophan, methionine and valine yields no negative effects on animal performance or nitrogen retention. The challenge of fatter carcasses generated by LP diets may be avoided by adoption of the NE system and balanced AA. However, concomitantly increased IMF content when pigs are fed LP diets could improve the eating experience required a novel proposition that how to increase IMF content in the case of thin backfat thickness. Thus, the underlying mechanism of how LP diets affect protein and fat metabolism should be elucidated, in order to provide a nutritional strategy improves the meat quality. With the development of biological fermentation technology, a greater range in CAA is available for feed use; therefore, a further reduction of dietary CP would be possible but routine implementation requires verification of the feasibility and practical significance.

## Abbreviations

AA: Amino acids
AID: Apparent ileal digestible
AMR: Antimicrobial resistance
BCAA: Branched-chain amino acids
BCFAs: Branched-chain fatty acids
CAA: Crystalline amino acids
CD4: Cluster of differentiation 4
CP: Crude protein
CSBM: Corn-soybean meal
DDGS: Distillers dried grains with soluble
DE: Digestible energy
EAA: Essential amino acids
ETEC: Enterotoxigenic *Escherichia coli*
*E. coli*: *Escherichia coli*
FCAA: Four crystalline amino acids
GIT: Gastro-intestinal tract
GSH: Glutathione
Hcy: Homocysteine
HP: High-protein
IFNγ: Interferon γ
IgG: Immunoglobulin G
IMF: Intramuscular fat
LP: Low-protein
ME: Metabolizable energy
mTOR: Mammalian target of rapamycin
mTORC1: Mammalian target of rapamycin complex 1
NCG: N-carbamylglutamate
NE: Net energy
NEAA: Non-essential amino acids
NO: Nitric oxide
P: Phosphorus
PUFA: Polyunsaturated fatty acids
PUN: Plasma urea nitrogen
PWD: Post-weaning diarrhea
SAA: Sulfur amino acids
SBM: Soybean meal
SCFAs: Short-chain fatty acids
SID: Standardized ileal digestible
SUN: Serum urea nitrogen
TID: True ileal digestible
TNFα: Tumor necrosis factor α
UPP: Ubiquitin-proteasome pathway
VLP: Very low-protein
WHC: Water holding capacity
